# Patient Involvement in the Lifecycle of Medicines According to Belgian Stakeholders: The Gap Between Theory and Practice

**DOI:** 10.3389/fmed.2018.00285

**Published:** 2018-10-11

**Authors:** Rosanne Janssens, Eline van Overbeeke, Lotte Verswijvel, Lissa Meeusen, Carolien Coenegrachts, Kim Pauwels, Marc Dooms, Hilde Stevens, Steven Simoens, Isabelle Huys

**Affiliations:** ^1^Department of Pharmaceutical and Pharmacological Sciences, KU Leuven, Leuven, Belgium; ^2^University Hospitals Leuven, Leuven, Belgium; ^3^Institute for Interdisciplinary Innovation in Healthcare, Université Libre Bruxelles, Brussels, Belgium

**Keywords:** patient involvement, decision-making, lifecycle of medicines, patient preferences, evaluation of medicines, reimbursement, medicines development, market access

## Abstract

**Background:** Patient involvement is often acknowledged as an important aspect of the lifecycle of medicines. Although different typologies exist, patient involvement has been described as the involvement of patients in decision-making regarding medicines. In view of the diversity of stakeholders and types of decisions in which patients might be involved, an in-depth understanding of these stakeholders' views toward involving patients in the lifecycle of medicines is essential.

**Methods:** Interviews and surveys were used to gain insights into the perspectives and experiences of Belgian healthcare stakeholders. Interviews (*n* = 22) were conducted with academics, hospital pharmacists and representatives from health insurance funds, the Belgian reimbursement agency, pharmaceutical industry and patient organizations. Interviews underwent a *framework analysis*. Surveys (*n* = 108) were completed by hospital visitors and analyzed descriptively.

**Results:** Despite an increasing amount of efforts to involve patients, interviewees labeled the level of actively involving patients as rather low and scattered across the different phases of the lifecycle of medicines. The main opportunities for patient involvement highlighted by interviewees were for: (i) informing early development decisions on which treatments to develop, (ii) clinical trial endpoint selection and (iii) clinical trial protocol design. However, remaining questions surrounding patient knowledge, and particularly how and which patients to involve represent important barriers toward implementing patient involvement in the lifecycle of medicines. Of survey participants, 77% indicated to be willing to participate in patient preference studies. Reasons for participating mentioned most frequently were “to improve development of treatments,” because “it is important to explore and listen to patient preferences” and “to have a voice as patients”.

**Conclusions:** The barriers identified in this study hamper transitioning patient involvement from theory to practice. Bridging this gap requires addressing the identified barriers and unresolved questions surrounding the right methodology for involving patients, the “right patients” to involve and means to increase patient knowledge. In order to do so, further research should focus on assessing the value of methods that allow to indirectly capture patients' perspective both in the context of development as well as in the context of evaluation.

## Introduction

Patient involvement is often acknowledged as an important aspect of the lifecycle of medicines (1–3), as reflected by numerous initiatives aiming to increase patient involvement. Examples include the PREFER^1^ and PARADIGM^2^ project under the *Innovative Medicines Initiative* (IMI). PREFER aims to establish recommendations toward different healthcare stakeholders on measurement and use of patient preferences. PARADIGM aims to provide a framework for structured patient engagement. Further, the public-private partnership *European Patients' Academy* (EUPATI) developed patient training modules and guidance documents (4). At the regulatory level, both the *U.S. Food and Drug Administration* (FDA) and the *European Medicines Agency* (EMA) attempt to facilitate patient involvement in their regulatory processes (5). In the U.S., the FDA has organized public workshops to inform the development of patient-focused guidance^3^, among other examples. In Europe, the EMA has put into place a number of mechanisms for increasing the patient's voice in regulatory activities, including the development of a framework for interaction with patients, consumers and their organizations^4^, the *Benefit-Risk Methodology Project* outlining *Conjoint Analysis* as a method for determining patient preferences and the conduct of the EMA *VALUE Study* to measure patient preferences for treatment outcomes (6, 7). EMA also publishes annual reports^5^ describing where and how patients contributed to EMA activities. In the post-marketing setting, a European database^6^ was set up to allow patients to report side effects online and in their native language.

Different typologies of patient involvement have been described in an effort to explain the term. Some authors differentiate between patient involvement on the micro-, meso- and macro-level, depending on whether the involvement impacts individual patients, a specific disease area, or resource allocation and priority decisions, respectively (8, 9). Others describe patient involvement through differentiating between “direct patient involvement” and “indirect patient involvement”, depending on whether patient involvement is operationalized through the participation of patient representatives in a group of decision makers or through indirect measurements of the patient perspective on the value of health innovations, respectively (10). The term patient involvement is often used interchangeably with the term patient participation; both terms have been used to refer to the involvement of patients in decision-making (10, 11). “Patient perspectives” and “patient preferences” are other (inter-)related terms. Whereas, “patient perspectives” have been used as an umbrella term to refer to patients' experiences, attitudes, beliefs and values, “patient preferences” have been defined in a more precise way (12); the FDA defines patient preference information as “*qualitative or quantitative assessments of the relative desirability or acceptability to patients of specified alternatives or choices among outcomes or other attributes that differ among alternative health interventions*” (13). In this study, “patient involvement” will be used as an umbrella term to refer to both: (i) direct patient involvement, via the participation of patients (or patient organizations) in discussions and (ii) indirect patient involvement, via the measurement and inclusion of patient perspectives on the value of health innovations via studies that allow to assess patient preferences, termed patient preference studies hereafter.

In the Belgian healthcare context specifically, the *Belgian Health Care Knowledge Centre* (KCE) assessed the acceptance of public and patient involvement among Belgian reimbursement stakeholders and formulated recommendations on how to involve patients and the public in decisions surrounding reimbursement and resource allocation (9). However, in view of the multitude and diversity of stakeholders and types of decisions in which patients might be involved during the lifecycle of medicines, an in-depth understanding of these stakeholders' views toward involving patients in the lifecycle of medicines is critical. Additionally, as the KCE report focusses on patient and public involvement at the reimbursement level and similarly, on the opinions of Belgian reimbursement stakeholders, an understanding of patient involvement *across* the lifecycle of medicines and among *a wide variety* of Belgian healthcare stakeholders seems lacking.

Therefore, this study aimed to map the experiences and perspectives of Belgian healthcare stakeholders toward patient involvement in the lifecycle of medicines. Although patient involvement might also take place at other levels of healthcare decision-making (e.g., during the medical consultation between patients and healthcare professionals), this study focussed on patient involvement from discovery up to and including post-marketing.

## Material and methods

### General design

Interviews and surveys were used simultaneously to gain insights into the perspectives and experiences of Belgian healthcare stakeholders since they allowed to explore the perspectives of different stakeholder groups and had a slightly different focus. The interviews were used to gain a thorough understanding of the experiences and reasoning of stakeholders within healthcare organizations (academics, hospital pharmacists and representatives from health insurance funds, the Belgian reimbursement agency, pharmaceutical industry and patient organizations) and addressed the broader topic of patient involvement^7^, including patient preferences^8^. The surveys allowed to get views from a large sample of hospital visitors (patients, informal caregivers and relatives) and focused on the topic of patient preferences^9^ as an example of a tool for patient involvement. Data were collected from October 2016 until December 2016. The study was approved by the Ethics Committee of the Faculty Pharmaceutical sciences at the University of Leuven, Belgium (reference: mp12721) (14).

### Interviews

#### Interviewees

Twenty-two semi-structured interviews^10^ were conducted with academics (*n* = 4), hospital pharmacists (*n* = 2) and representatives from health insurance funds (*n* = 3), the Belgian reimbursement agency (*n* = 1), pharmaceutical industry (*n* = 8) and patient organizations (*n* = 5). Invited interviewees were considered able to provide guidance with regard to the research aims (purposive sampling). An initial sample of potential interviewees was suggested by the co-authors. In total, 65 persons were sent an invitation.

#### Interviews

An interview guide was developed based upon a literature review and consisted of eight open-ended questions on the following three topics: (i) the value of patient involvement (including patient preferences) in the different phases of the lifecycle of medicines and for different healthcare stakeholders, (ii) the current level of patient involvement (including patient preferences) in the lifecycle of medicines and (iii) the currently used methods for measuring patient preferences in the lifecycle of medicines (Supplemental Material 1). The interview was piloted with two persons, including with one researcher involved in the study (MD). Since MD was not involved in the design of the interview guide nor the further analysis of the interviews, both of the pilot interviews were taken up in the analysis. The interviews were conducted face-to-face or via WebEx and in the mother tongue of the interviewee (Dutch) by three researchers (CC, LM, LV). Written informed consent was obtained prior to the interview. The interviews lasted between 20 and 60 min, were audio recorded and transcribed verbatim. Interviewees were not remunerated.

#### Analysis

Characteristics of the interviewees were summarized descriptively. Interviewees were given a code referring to their primary affiliation and ID number. The interviews were analyzed using *framework analysis* (15) (Table 1; Supplemental Material 2).

**Table 1:** Stages of framework analysis.

Familiarization	The interviews were transcribed verbatim by three researchers (CC, LM, LV) and the transcripts were thoroughly read by the researchers involved in the analysis (RJ, CC, LM, LV).
Identifying a thematic framework	Three researchers (CC, LM, LV) independently coded the same two transcripts before meeting to develop an initial list of codes, i.e., the initial coding framework. The initial framework was further discussed, refined and agreed upon together the remainder of the researchers involved in the analysis (EvO, RJ).
Indexing	The transcripts (*n* = 22) were divided equally among three researchers (CC, LM, LV) who applied the codes to the data, using the qualitative data analysis software NVivo (11th edition, QSR International).
Charting	NVivo enabled the creation of a framework matrix, which was subsequently exported to an Excel file. In the matrix, the interviewee codes were listed in the y-axis and the codes from the final framework on the x-axis. The coded text was summarized for each code and interviewee in the corresponding cell by one researcher (RJ).
Mapping and interpretation	Using the Excel framework matrix created in stage 4, one researcher (RJ) searched for themes, associations, concepts and explanations in the data. This process was guided by the research aims and a careful analysis of what was in the data. Interpretations were made by reviewing the matrix and making associations within codes and interviewees, as well as between codes and interviewees. Whenever the data was rich enough, the interpretations generated in this stage went beyond the description of a particular interviewee to the explanation of potential reasons or beliefs of multiple interviewees.

### Surveys

#### Survey participants

Participants were approached by three researchers (CC, LM, LV) in the hall of the hospital UZ Leuven, Belgium during October 2016. Participants included patients, informal caregivers and relatives. Participants were included if they: (i) were aged between 18 and 85, (ii) comprehended Dutch, and (iii) were able to understand and respond to the questions of the survey. Participants were not remunerated.

#### Survey

An anonymous, self-administered, paper-based survey was developed based upon a literature review and consisted of 10 closed and open questions about the following topics: (i) the importance of patient preferences in the different phases of the lifecycle of medicines, (ii) the current use of patient preferences in the different phases of the lifecycle of medicines, and (iii) the experience of the participant with patient preference studies^11^ and their willingness to participate in such studies (Supplemental Material 3). Prior to filling in the survey by themselves, the study was explained and participants were given time to read the information sheet. Above the questions in the survey, a definition of patient preferences^12^ was given, but it was not verified whether patients had read and understood this definition. Questions containing terms related to medicines development were explained in between brackets. Participants had the possibility to ask questions when facing difficulties while they completed the survey.

#### Analysis

Data was analyzed anonymously in Excel using descriptive statistics. No statistical testing was performed.

## Results

### Interviews

The interviews results section is structured according to the main themes identified during the final stage of the framework analysis. Codes between brackets and following the quotations refer to interviewees' characteristics and are formatted in the following way: (i) stakeholder group (Table 2; Supplemental Material 4), (ii) ID number of the interviewee.

**Table 2:** Demographics of interviewees.

**Demographics**	**Stakeholder group code**	Interviewees^*^ (*n* = 23)	
		***n***	**%**
**SEX**			
Male		13	57
Female		10	43
**NATIVE LANGUAGE**			
Dutch		23	100
**STAKEHOLDER GROUP**			
Academic, active as physician	A	4	17
Health insurance fund	B	3	13
Belgian reimbursement agency^**^	C	1	4
Pharmaceutical industry	D	8	35
Patient organization	E	5	22
Hospital pharmacist	F	2	9

* *One interviewee preferred to take part together with one extra interviewee, resulting in a total amount of 23 interviewees and 22 interviews*.

** *Belgian reimbursement agency (National Institute for Health and Disability Insurance National Institute for Health and Disability Insurance, NIHDI)*.

#### Demographics of interviewees

Of the invited 65 persons, 35% accepted the invitation. Others did not answer or had no time or interest to participate. The majority of interviewees were male (57%) and employees of pharmaceutical industry (35%). Four interviewees (17%) have multiple affiliations and therefore belong to multiple stakeholder groups. For the stakeholder group and codes following the quotations of the interviewees, interviewees were assigned to only one stakeholder group, based upon their primary employment or affiliation.

#### Opportunities and barriers related to patient involvement in the lifecycle of medicines

**Figure 1:**
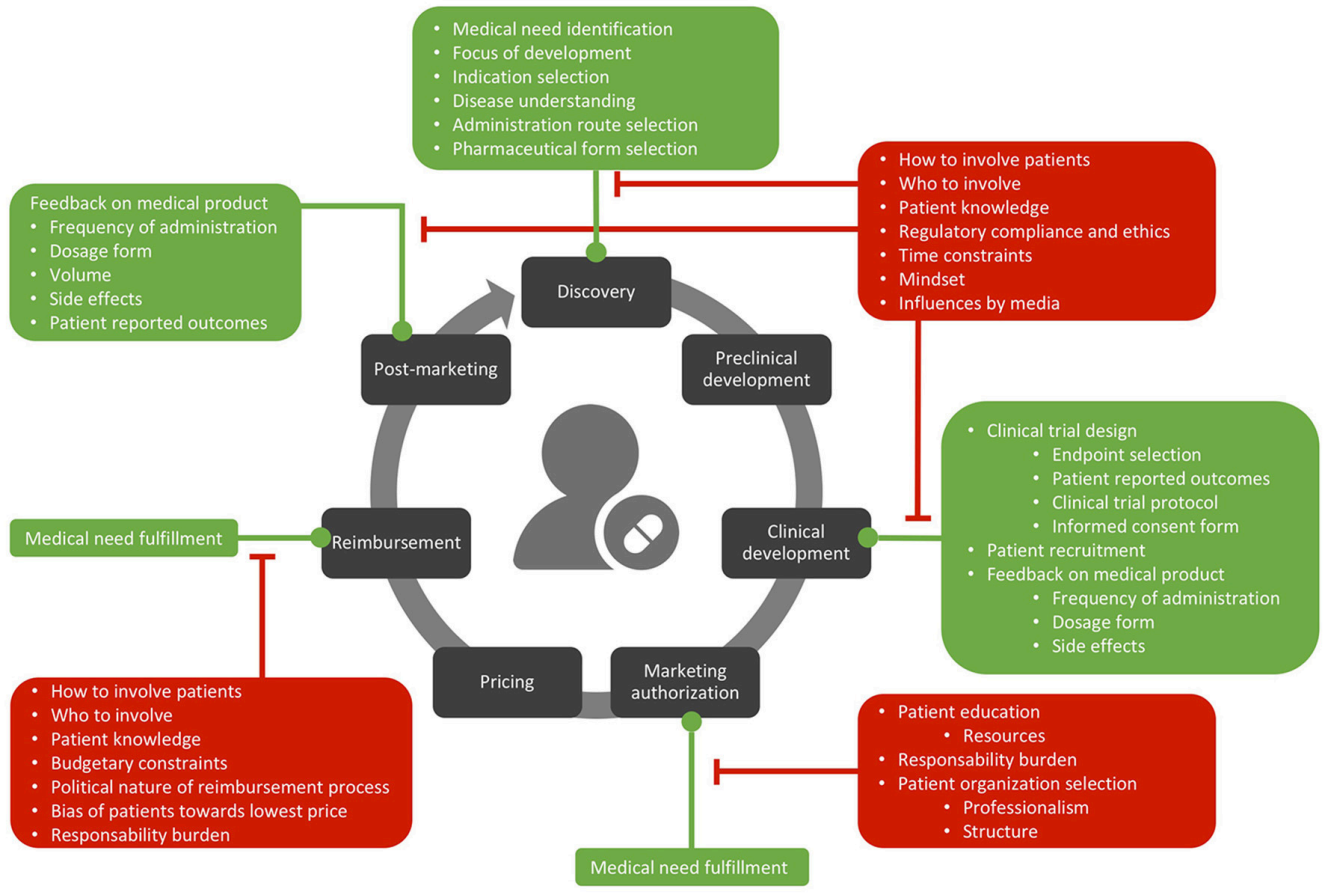
Opportunities (in green) and barriers (in red) related to patient involvement in the lifecycle of medicines.

The opportunities and barriers interviewees described are graphically presented in Figure 1 and described at length below.

##### Opportunities and barriers in medicines development and post-marketing

Opportunities in medicines development and post-marketing

**Discovery**. Several interviewees (A,C,D,E) underlined the potential role of patient involvement in early medicines development. More specifically, interviewees argued how patient input should be used to: (i) identify medical needs, (ii) inform decisions on what medicines would be relevant to develop, (iii) acquire a deeper understanding of the disease, (iv) inform early decisions on administration routes and (v) inform decisions on the targeted indications (Figure 1): “This *will even determine ‘we are going to develop a product that could be used in hypertension, but also in heart failure'*” (D13). A patient representative reasoned that “*if patient needs are not taken into account from early on, then it becomes uncertain whether the product eventually marketed will answer these patient needs*” (E23). Similarly, involving patients during early development would lead to a bigger return on investment according to several interviewees, since “*they will not make profit if they put something on the market that has no added value and where there is no demand for*” (D14).

**Preclinical development**. In contrast to the potential role of patient input in informing early development, the role of patient involvement in preclinical development was labeled minimal by several interviewees, as this research often takes place in animals or cell lines and is very technical.

**Clinical development**. Several interviewees (A, C, D, E) described the benefits of patient involvement for: (i) the selection of endpoints, (ii) the development of the clinical trial protocol and informed consent, and (iii) patient recruitment. An academic stated that using patient views for clinical endpoint determinations would counter the current approach of clinical trials in phase 2 and 3, which is currently “*focused on reaching a statistical difference*” (A2). This view was shared by a patient organization representative, arguing that asking patient views on primary clinical trial endpoints could help to “*shift the focus of the clinical trial*” (E22). Some interviewees argued that having patient views on the acceptability and feasibility of the clinical trial design specifically in rare diseases would be beneficial since: (i) physicians involved in clinical trial design are often not familiar with the disease because of the rarity of the disease, and (ii) it would avoid dropout during the trial itself due to the clinical trial being too exhausting for patients: “*The rarer the disease, the more important that patients themselves are involved through their organizations because it has been proven that a lot of studies have failed when patient input was not taken into account sufficiently*” (E23).

Another potential opportunity within the design of clinical trials mentioned by a patient representative was in the set-up of inclusion and exclusion criteria of clinical trials, as these criteria were labeled as “*a bit too unrealistic, especially in the case of ALS*^13^*. For most clinical trials, you have to have a vital lung capacity of 70%, if you blow 68 twice you are excluded from the study. Unrealistic because ALS puts itself immediately on the lungs*” (E19). The view that inclusion criteria are extremely narrow was shared among interviewees from industry and patient organizations. An industry interviewee explained however that changing in-and exclusion criteria during clinical trials according to the needs of one patient would “*mess up your whole research*” (D16).

While some academics minimized the role of patient involvement in phase 1 clinical trials, arguing that these are mostly performed on healthy volunteers, other interviewees mentioned that patients and patient organizations could provide input on the clinical trial protocol, to increase the readability of informed consent forms and recruitment of healthy volunteers. A hospital pharmacist argued that during the phase 1 trial itself, patients could provide input on frequency of administration, dosage form and their tolerance of side effects.

However, the involvement of patients in clinical trial design was not supported by some interviewees because of the level of required expertise and time restrictions. Moreover, several interviewees were unclear how and which patients could be involved (see “Barriers in medicines development”).

Finally, engaging with patient organizations to enhance patient recruitment in phase 2 and 3 clinical trials would speed up medicines development according to some academics, which is especially relevant for clinical trials conducted with patients with rare diseases according to an academic, since patient organizations have good connections with patients themselves and since recruitment here is often challenging due to the rarity of the disease.

**Post-marketing activities**. According to some interviewees (E, D, F) using patient views collected in the post-marketing setting for adapting aspects of administration, such as frequency, volume and form of administration, in early development would enhance patients' compliance to the medicine. An industry interviewee further elaborated that a bad compliance not only affects patients' health, but also increases societal burden, since unused medicines are a “*waste of money*” (D16) and may inhibit patients to return to work.

**Post-marketing activities related to safety (post-marketing surveillance)**. Although interviewees from all stakeholder groups agreed that the current system for reporting side effects is improvable, the opinions on whether or not this should be done by increasing the role of patients were mixed among interviewees. While some interviewees argued that allowing patients to report side effects directly to the *Federal Agency for Medicines and Health Products* (FAMHP) via their website would lead to more side effects being reported, others feared that this would endanger the reliability of reported side effects and increase the managerial burden for the FAMHP and pharmaceutical company who have to follow-up on each side effect. Likewise, some interviewees emphasized that this would raise a need for a filtering system. Several interviewees concluded that because of these difficulties, the responsibility of reporting side effects should remain with physicians and pharmacists, rather than patients. Further, the idea of physically involving patients on a more managerial level in the procedures of post-marketing surveillance was labeled “*absurd*” by an industry interviewee, who characterized the pharmacovigilance phase as “*a strictly regulated system*” (D13). Besides the role of patients themselves, the role of patient organizations in post-marketing surveillance was also discussed. A patient representative did not foresee a role of patient organizations in post-marketing surveillance, reasoning that “*the more technical the issue, the less important it is that patient organizations are involved*” (E23). On the other hand, some interviewees thought patients could be involved “*to see how to improve this system*” (D12), emphasizing that in this case, questions arise around how this involvement would be implemented practically.

Barriers in medicines development and post-marketing

**How to involve patients in development**. Several interviewees were unclear about how to operationalize patient involvement in development and post-marketing (Figure 1). Some industry interviewees simply stated that involvement of patients in medicines development is complex or not always feasible. Several interviewees reflected on this issue and stressed the importance of “*doing it the right way*” (D13).

**Who to involve**. Another question several interviewees raised but could not answer themselves, was the question on “who to involve”: “*If you would say ‘the patient', who is that then?*” (D13). Interviewees held diverging views on whether or not individual patients and/or patient organizations should be involved. Some interviewees (D, E) suggested the involvement of patient organizations rather than individual patients because: (i) this would avoid bias toward the preference of one specific patient: “*a direct link between the ‘therapy for myself' and the work results in bias*” (E23), (ii) it would eliminate barriers of compliance and ethical aspects, (iii) it is not feasible to reach every individual patient: “*It is simply way too difficult to consult with everybody and opinions might be diverging so I think that (patient) organizations can have an important role in this regard*” (E19). On the other hand, involving patient organizations was also criticized because: (i) the impact of patient organizations on decision-making is subject to differences in the level of professionality, knowledge and size of patient organizations across different disease areas (B, D), (ii) questions on whether patient organizations are representative for patients, since patient organizations often do not reach all patients, have a political agenda and often depend on industry, (iii) the question of whether disease-specific vs. umbrella patient organizations should be involved; some interviewees underlined that disease specific patient organizations should be involved when the discussion involves a disease-specific topic. Finally, an industry interviewee suggested the combination of involving both of patient organizations and individual patients to have a “*global picture*” (D12).

**Patient knowledge vs. the complexity of development**. Several interviewees doubted whether patients have sufficient knowledge and competences to contribute to decision-making. An industry interviewee explained that although patients can contribute indirectly, e.g., via questionnaires, to decide on whether or not to include *Patient Reported Outcomes* (PROs) in clinical trials, direct involvement in clinical trial design is difficult since this requires specific technical knowledge. Similarly, some interviewees stressed the importance of educating patients prior to asking their input on development related aspects. One patient representative stressed that to be able to give correct information, patients need to understand what their input will be used for: “*Patients who do not understand what will happen with their input can also result in bias and they can give wrong information just because they do not know about the context*” (E23).

**Regulatory compliance and ethical aspects**. Although several interviewees (A, D, F) believed direct contact between pharmaceutical companies and patients is not allowed, an industry interviewee mentioned that industry can approach patients if the contact does not have a commercial purpose. Nonetheless, interviewees (A, D, F, E) emphasized that ethical and regulatory compliance aspects complicate direct patient involvement: “*It is not so easy for companies to work with patients*” (E23). An industry interviewee reasoned because of the difficulty of directly engaging directly with individual patients, industry prefers asking input from patient organizations. Another industry interviewee argued that because of this difficulty, patient input should be gathered via methods that allow to indirectly ask patient views.

**Tight timeline of development**. Some interviewees (A, D) explained that involving patients during medicines development is difficult because it would make the process more complex and time-consuming: “*If there is something that the industry does not want, then it is that it takes longer*” (A6).

**Current mind-set**. An industry interviewee expressed that transitioning into a new way of medicines development where patients are actually involved, would require time and a change in mind-set: “*If one now would suddenly say: ‘we are going to make the patient a major stakeholder in the entire development process of medicines'. People are not going to know what to say*” (D15).

**Influences by media**. Several interviewees (B, D, F) mentioned that patients are often influenced or manipulated by media, which was criticized for being an unreliable information source.

##### Opportunities and barriers in the marketing authorization procedure

When reflecting on the role of patients and patient organizations in the national marketing authorization procedure, the following barriers were mentioned: (i) one interviewee (B) feared that patients would only speak from their own perspective and their disease, whereas marketing authorization (and reimbursement decisions, see “Barriers, who to involve”) also impacts healthy citizens and patients in other disease areas, (ii) the level of knowledge required to participate in decision committees: “*These are difficult discussions, you cannot put a person there without any knowledge and expect that this person at the end of the discussion can add something of value*” (E22) and similarly, (iii) the need for educating patient organizations and resources needed to do this (D), (iv) the difficulty of finding patient organizations wanting to participate due to the responsibility inherent with this task, (v) the question about whether disease-specific or umbrella patient organizations should become involved, and (vi) one interviewee (D) added that the impact of Belgian patient organizations is often limited because of a lower level of professionalization and structure as compared to foreign patient organizations (Figure 1). This heterogeneity among patient organizations across countries was confirmed by a patient representative. The representative stated preferring working with patient organizations who have in their management board patients or caregivers: “*At least 60 percent has to be directly involved (with the disease), otherwise it gets taken over by professionals*” (E19).

Rather than having a decisive role during the marketing authorization procedure itself, a sickness insurance fund representative foresaw a role for citizens in determining whether the medicines being evaluated by the FAMHP and the *National Institute for Health and Disability Insurance* (NIHDI) answers a medical need (see “Opportunities in reimbursement”).

##### Opportunities and barriers in pricing of medicines

Several interviewees from (A, D, E) were unsure about the value of direct patient involvement in pricing discussions, arguing that these discussions require specific expertise and patients and patient organizations would have the sole objective of wanting the lowest price. An industry interviewee differentiated between having patients co-decide on pricing vs. having their indirect input on pricing via indirect research; while the former was described as difficult since pricing discussions are complex and context-specific, the latter was found to be desirable in cases with low or limited reimbursement.

##### Opportunities and barriers in reimbursement

Opportunities in reimbursement

Although several interviewees (B, D) foresaw an evolution toward a more patient centric reimbursement procedure, mixed opinions were expressed regarding directly involving patients and patient representatives in the reimbursement discussion itself. Reasons why interviewees doubted the direct involvement of patients revolved around the remaining questions of how and which patients to involve, patient knowledge and budget issues (see “Barriers in reimbursement”).

Rather than involving them during the reimbursement discussion itself, some interviewees felt that patient involvement in early development decisions “*would immediately allow for the patient perspective to be captured during the reimbursement discussion*” (E20), thereby increasing the chance of getting the medicine reimbursed as “*the criterion of ‘meeting the patient requirements' is automatically fulfilled*” (E23). Mirroring positive opinions toward involving patients for early development determinations of medical need, some interviewees explained that input from citizens and patients would be beneficial in assessing whether the medicine answers a medical need: “*In fact we as a society must be able to say that we have that societal need (…) And I think that is something that citizens can be involved with*” (B5) (Figure 1).

##### Barriers in reimbursement

**How to involve patients**. Several interviewees (A, B) were uncertain about how patient perspectives could be reflected in the reimbursement discussion and underlined the complexity of this matter together with the importance of having “*the right methodology*” (B5) (Figure 1). In this regard, some interviewees reflected on the Soliris® case, warning that this case should be viewed as an example of how it should not be done, as in this case, according to an academic, patients were used to “*emotionally blackmail*” (A7) the government^14^. Some interviewees from health insurance funds suggested to use the list of unmet medical needs (see “Current Landscape of Patient Involvement,” reimbursement) as an incentive toward companies by increasing their chances on reimbursement if they develop medicines answering a medical need that scores high on the list. Some interviewees suggested the use of methods that enable the inclusion of patient perspectives without directly involving them in the discussion, such as *Multiple-criteria Decision Analysis* (MCDA), which enables the measurement of patient preferences for characteristics of medicines, and the development of PROs and *Patient Reported Experience Measures* (PREMs).

**Who to involve**. Some interviewees (A, C, B) feared that patients and disease specific patient organizations would only speak from their own perspective and their disease, thereby creating a risk of disparity among disease areas: “*Their disease is very important to them and that can be at the expense of something else*” (B4).

Regarding the involvement of patient organizations, some interviewees (B) reasoned that funding of patient organizations by pharmaceutical companies endangers their independency. One interviewee concluded that because of doubts on objectiveness and neutrality, patient organizations should not have a voting right in reimbursement decisions: “*The moment you have to make the weighting, the choices, that you have a more global view, independent, neutral vision*” (B5). The same interviewee however suggested to take into account input from patient organizations in preparation of dossiers being discussed during reimbursement discussions. A patient representative suggested to involve umbrella patient organizations to ensure a more neutral and rational discussion.

**Patient knowledge vs. the complexity of reimbursement**. Several interviewees (A, B, D) underlined the importance of education, knowledge and experience as a prerequisite for having a voting right in reimbursement decisions and worried about the level of knowledge of patients to be able to contribute to the discussion.

**Budgetary constraints**. Some interviewees (A, D, E) mentioned that patient input in medicines reimbursement needs to be considered in view of budgetary and economical aspects: “*We are dealing with the economic pressure at the moment, there are also many economizations, (…) of course they have to happen somewhere*” (D12).

**Other** described barriers associated with involving patients in reimbursement included: (i) the fact that reimbursement is a politically driven process in which patients might not be able to contribute (A), (ii) patients and disease specific patient organizations would be biased toward paying as little as possible (D, E), and (iii) the burden and responsibility of the tasks associated with having a voting right within the reimbursement decision committee (D).

#### Current landscape of patient involvement in the lifecycle of medicines

**Across the lifecycle of medicines**. Although references were made to an increasing number of examples and efforts coming from industry, regulatory and reimbursement stakeholders, interviewees (A, D, F) labeled the level and approaches of actively requesting patient input during medicines development as rather low and scattered across different phases of the lifecycle of medicines. Moreover, the level of patient involvement was described to be variable across: (i) pharmaceutical companies (D), (ii) countries (A, D), since patient organizations in a specific country (e.g., Germany) are better organized or in some countries, the headquarters are localized that consult with patients through meetings and market research, and (iii) whether the involvement is directly or indirectly via other methods. While the latter was described as being already pursued by pharmaceutical companies, the former was described as “*more complex*” (D13).

**Discovery**. While some industry interviewees stated that pharmaceutical companies currently “*listen*” (D14) to patients as early as possible, the level of patient involvement in the earliest phases was labeled as “*rather limited*” (D12) by another industry interviewee. Interviewees (A, B, C, D) criticized the current paradigm for early development for being driven by a supply-instead of demand driven approach. This approach is also reflected in current pharmaceutical research and selection of endpoints in clinical trials, which were criticized by some interviewees for not always being patient-focused: “*In our case specifically (Huntington), science is mainly interested in the uncontrolled movements, to limit them (…) But for patients that is not the biggest problem, their biggest problem is the character changes*” (E20).

**Clinical development**. Whereas, some interviewees mentioned that the level of patient involvement is low or non-existing and purely consists out of patients participating as research subjects in clinical trials, other interviewees explained that pharmaceutical companies increasingly consult with patients and patient organizations to verify whether the clinical trial answers their needs, e.g., via asking questions on the administration form and relevance of the endpoints. Industry also seems to increasingly connect with patient organizations to enhance patient recruitment for phase 2 and phase 3 clinical trials according to some interviewees. However, this was labeled by a hospital pharmacist as making abuse of patient organizations “*to find test subjects, for example in rare diseases they approach patient associations*” (F9).

**Clinical trial evaluation and marketing authorization**. While an industry interviewee stated that the level of patient involvement in the Belgian marketing authorization procedure is limited, some interviewees (B, D, E) explained that a recent initiative by the FAMHP led to the presence of a patient representative in the commission tasked with evaluating clinical trials and advising the minister on the national marketing authorization. Regarding patient involvement on the European level, while some interviewees described that EMA currently “*looks at the patient perspective*” (E19), an industry interviewee had the impression that in practice, patients are not at all involved. A patient organization representative mentioned their interaction with EMA and role in verifying whether clinical trials are patient oriented.

**Pricing**. Interviewees across stakeholder groups described that patients are not directly involved in current pricing discussions in Belgium. Some industry interviewees mentioned that the patient voice on aspects of pricing is sometimes gathered indirectly via studies preceding the price setting, e.g., via willingness-to-pay studies. Further, an academic stated that some patient organizations try to influence the price setting by lobbying.

**Reimbursement**. While some interviewees stated that patients are currently not present during the reimbursement discussion, others described that the reimbursement system recently changed to allow for patient representatives functioning as observers during the discussions. This was positively experienced as “*some things will be discussed differently when a patient is present*” (A2). Some interviewees (B) explained that currently, health insurance funds try to represent the payer of healthcare in reimbursement discussions, including patients but also healthy citizens. However, industry and academic interviewees doubted the ability of health insurance funds in representing patients, since they “*do not always have sufficient insight into the concrete needs of patients*” (B5). An industry interviewee mentioned that patients' perspectives are reflected indirectly in the reimbursement discussion via the utilization of the *Quality-Adjusted Life Year* (QALY) measure.

Interviewees mentioned recent initiatives that explore how patient and citizen perspectives can be captured in reimbursement discussions. The initiative mentioned most frequently was the organization of citizen meetings to gather their views on the relevance of reimbursement decision criteria (A, C, D). Interviewees further explained that these criteria were subsequently used to rank unmet medical needs and that this list informs reimbursement decisions. Some interviewees (A, C) criticized that the indications on the list are currently only being suggested by pharmaceutical companies: “*It are the companies that suggested their medicines, saying we have this medicine ready, what do you think? This is not driven by a demand*” (B5). An academic further doubted: (i) whether the results from these citizen meetings are generalizable to a wider population and (ii) whether the meetings informing the criteria should not also have been with patients, instead of only citizens.

According to some interviewees (F, D), certain patient organizations try to speed up the reimbursement discussion by lobbying. According to an industry interviewee however, the extent to which patient organizations can impact reimbursement via lobbying depends on their competences, connections and overall size.

**Post-marketing activities**. According to some interviewees, patient input is currently mostly gathered in this phase; according to an industry interviewee this input is gathered indirectly (e.g., via PROs) and often results from a commitment between the pharmaceutical company and the Belgian reimbursement authority to demonstrate the promised added value of the medicine in real life. An academic criticized the collection of patient input in the post-marketing setting for being a marketing stunt. The same interviewee further explained that the questions in the surveys used in this setting are often posed in a biased way, supporting the claim of the company. According to some industry interviewees, pharmaceutical companies increasingly try to capture patients' experiences with their illnesses (e.g., via organizing workshops) with the aim of understanding their experiences and needs to eventually “*be able to respond to those needs*” (D11).

**Post-marketing activities related to safety (post-marketing surveillance)**. Some interviewees explained the recent change that enables patients to report side effects directly to the FAMHP. However, interviewees from all stakeholder groups criticized this system for not being able to capture side effects sufficiently and more specifically that currently, there are too few side effects being reported by physicians and patients. Some interviewees attributed the lack of reporting to: (i) the amount of time that this requires of the physician, (ii) the fact that patients are not aware they can report themselves and (iii) the complexity of self-reporting via the website of the FAMHP: “*The reporting procedure is too difficult to do, which results in the fact that patients who click on this link, say: ‘never mind'*” (E22). In contrast, some interviewees were positive about the recent change enabling the reporting of side effects by patients (see “Opportunities in medicines development and post-marketing, post-marketing activities related to safety”).

### Surveys

Whereas interviews addressed the general topic of patient involvement (including patient preferences), surveys focused on the topic of patient preferences as an example of a tool for patient involvement. No statistical testing was performed on survey responses; therefore, they are not to be interpreted as statistically significant. All survey participants answered all questions, except for the open question asking about why they would or would not participate in patient preference study. Eighty-seven survey participants (81%) responded to this question.

#### Demographics of survey participants

Among 108 survey participants, most were female (55%), lived in Belgium (97%) and did not mention a disease (63%). Included patients belonged to a large variety of disease areas. The most represented disease area was cardiovascular diseases (Table 3). The mean age of survey participants was 47.

**Table 3:** Demographics of survey participants.

**Demographics**	**Survey participants (*n* = 108)**	
	***n***	**%**
**SEX**		
Male	49	45
Female	59	55
**AGE**		
18–25 years	24	22
26–35 years	7	6
36–45 years	14	13
46–55 years	24	22
56–65 years	20	19
66–75 years	15	14
76–85 years	4	4
**NATIONALITY**		
Belgian	105	97
The Netherlands	3	3
DISEASE AREA^*^		
Respiratory tract disease	4	4
Cardiovascular diseases	13	12
Injuries	7	6
Cancer	7	6
Hormonal diseases	2	2
Anxiety disorders	1	1
Musculoskeletal diseases	1	1
Female disease of the urinary and reproductive systems and pregnancy complications	1	1
Digestive system diseases	5	5
No disease mentioned	70	65

* *Some survey participants indicated multiple diseases, resulting in a total percentage of more than 100%*.

#### Importance of incorporating patient preferences

**Figure 2:**
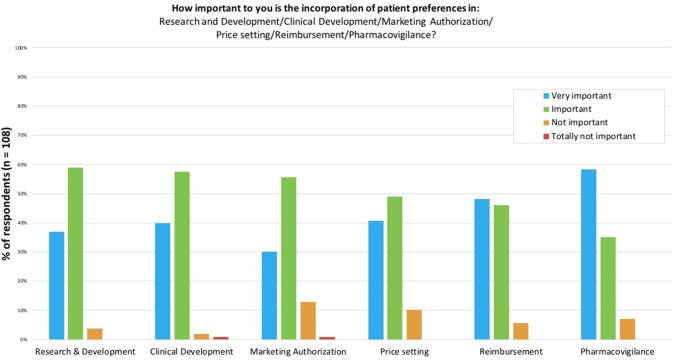
The importance to survey participants of incorporating patient preferences in the different stages of the lifecycle of medicines. Participants were asked to score the importance of incorporating patient preferences per stage in the lifecycle of medicines on a scale of “Very important” to “Totally not important”.

In the first part of the survey, participants were asked about the importance of measuring and using patient preferences during the different phases of the lifecycle of medicines (Figure 2): *Research and Development* (R&D), clinical development, marketing authorization, price setting, reimbursement and pharmacovigilance. Around 90% of participants considered the use of patient preferences “important” or “very important” for all phases. Pharmacovigilance was the only phase for which the majority of participants (58%) found the use of patient preferences “very important”. Use of patient preferences in R&D, clinical development and marketing authorization was considered “important” by the majority of participants (59, 57, and 56%, respectively). The phase that reached the highest percentage of participants (14%) finding the incorporation of patient preferences “not important” or “totally not important” in decision-making at this stage was marketing authorization.

#### Current use of patient preferences

Subsequent to the questions on the importance of patient preferences at the different stages, participants were asked to estimate the current level of use of patient preferences. Most participants thought that patient preferences were not used a lot in the lifecycle of medicines. Patient preferences were estimated to be “moderately” used by 36% of participants, and “little” to “too little” by 33 and 18%, respectively. Participants were asked if they already participated in patient preference studies. Of participants, 9% answered that they had already participated in a patient preference study. However, when participants explained the study they took part in, it became clear that they confused the term “patient preference study” with the term “clinical trial”. As a result, it is highly likely that none of the participants had participated in a patient preference study.

#### Willingness to participate in patient preference studies

Participants were asked to indicate whether they were willing to participate in patient preference studies or not. Of participants, 77% were willing to participate and 23% were not. Subsequently, participants were asked in an open manner why they would, or would not, want to participate in a patient preference study. A total of 87 participants (81%) responded to this question. Among the responding participants, 56 participants mentioned clear reasons for participating in patient preference studies and 14 participants mentioned reasons for not participating. Several participants indicated more than one reason. Reasons for participation mentioned most frequently by the 56 participants were “to improve development of treatments,” “it is important to explore and listen to patient preferences,” and “to have a voice as patients”. Other frequently mentioned reasons included “to contribute to society,” “to improve outcomes for the next generation of patients” and “it is important that patients agree with the medication they take”. Reasons for not participating in patient preference studies mentioned most frequently by the 14 participants not willing to participate were “no time” and “I know too little about it”. In addition, participants were asked in an open manner how patient preference studies should be organized. Of the responses from 38 participants, the most prevalent suggestions were “Via Health Care Professionals” (29%) and “Online” (29%). There were more participants that preferred “Surveys” (21%) than participants that preferred “Meetings and group discussions” (16%) or “Interviews” (13%).

## Discussion

Through conducting interviews and surveys, this study provides a detailed understanding of the different perceptions among Belgian stakeholders toward patient involvement in the lifecycle of medicines.

### Opportunities and barriers related to patient involvement in the lifecycle of medicines

The main opportunities for patient involvement in medicines development highlighted by interviewees in this study were for directing early development (e.g., identifying unmet medical need and informing decisions on which treatments to develop) and during clinical development (e.g., selecting clinical trial endpoint selection and informing protocol design; Figure 1). These opportunities have been described in literature (5, 17, 18) and were also identified in a qualitative interview study conducted by Lowe et al. (1) aiming to gain a better understanding of the present state of patient involvement in development. Smith et al. (19) used surveys to explore attitudes toward engaging patient organizations and found similar results; survey respondents valued the importance of involving patient groups in clinical research and patient group respondents valued their contributions to research protocol development. However, they also found other opportunities for the involvement of patient organizations, including in the interpretation of study results (19). Young et al. (20) performed a scoping review to identify opportunities of patient involvement specifically in the orphan lifecycle of medicines and categorized them into 12 themes. Among these, informing clinical trials and PRO were opportunities also revealed in the present study. Young et al. (20) however also identified opportunities outside the scope of the current study, including the contribution of patients to patient registries, conferences, workshops, patient care and support.

In contrast to early development and clinical development, it was unclear to interviewees in the current study how patients could contribute to preclinical development, as this phase is characterized by technical aspects and often studies are conducted on animals. Interviewees were also uncertain about the role of patient involvement in pricing discussions, as some argued that the technical nature of pricing discussions and the bias of patients toward wanting the lowest price would preclude patient involvement at this particular level. Additionally, in the post-marketing surveillance setting, interviewees held diverging opinions on whether or not improving the current Belgian system for reporting side effects should be operationalized by increasing the role of patients. Some interviewees argued that patients do not know they can report side effects via the website of the FAMHP and that this website is too difficult to use. Moreover, some interviewees questioned the reliability of these reported side effects. This latter finding somewhat contrasts with the findings described by Rolfes et al. (21) who found that the quality of adverse events reported by patients was comparable to that of healthcare professionals. Rolfes et al. (21) however also describe that a large part of the general public is not aware of the possibility to report side effects and highlight the importance of raising public awareness of the existence and purpose of pharmacovigilance. The present interviews also revealed several barriers connected with operationalizing patient involvement in medicines development, namely the question on how and which patients to involve, as well as aspects surrounding patient knowledge, regulatory compliance, timing constraints, the current mind-set and influences by media (Figure 1). A study by Parsons et al. (22) that aimed to discover beliefs of industry representatives on involving patients in Research & Development (R&D) found some similar barriers, as the study concluded that many interviewees were uncertain about when, how and which patients to involve. Furthermore, some interviewees in the study by Parsons et al. (22) did not see a role for patients being more actively involved, and the authors conclude that “*in such situations, cultural changes regarding how patients' roles in their healthcare are viewed may be needed*”. The barrier of who to involve is also confirmed by Lowe et al. (1), where interviewees expressed concerns about patient representativeness in medicines development discussions and by Caroll et al. (23), who identified the challenge of finding representative and appropriate patients for engaging patients in clinical research though interviewing and surveying research scientists. Caroll et al. (23) also identified the concern about patient knowledge and the need for education and cultural change as a challenge for patient involvement in clinical research. In the present study, when talking about the actual implementation of patient involvement in development, interviewees also underlined the tension between the technicalities inherent to this process and the level of knowledge this would require on behalf of patients. The barrier of patient knowledge was also found by Lowe et al. (1), reporting the concern of interviewees on the ability of patients to participate in medicines development conversations, and by Parsons et al. (22), where industry representatives believed that patients and the public's lack of knowledge in medicines R&D among others, are key challenges to increasing patient involvement.

In the present study, although several interviewees foresaw an evolution toward patient and citizen input in Belgian reimbursement procedures, some barriers mentioned in the context of medicines development also seemed to apply in the context of reimbursement; namely the unresolved questions on *who* and *how* to practically implement patient involvement and the importance of education, knowledge and experience as a prerequisite for having a voting right in reimbursement decisions. A qualitative study by Young et al. (24) explored ways in which Canadian patients with rare diseases and their families would like to be involved in the lifecycle of medicines and identified that patients themselves prioritized their involvement in reimbursement decisions, but that “*the ideal mechanisms for providing this input have yet to be determined*”. Further, the Delphi study conducted by the KCE to assess the acceptability of public and patient involvement in healthcare decisions to Belgian stakeholders concluded that although Belgian stakeholders agree on the importance of involving patients and the public in healthcare decisions, there seemed to be no openness to a complete overhaul or major revision of the present system and changes should take place inside current structures (9). Additionally, most respondents of the Delphi study had no idea of how the value of patient participation could be created in practice, and with which techniques and type of representation. The findings by Young et al. (24) and the Delphi study (9) seem to align with doubts interviewees expressed in the present study on how patients should become more involved in reimbursement discussions. Moreover, the remaining question of which patients to involve in reimbursement discussions also seems to be reflected in the risks identified by the KCE study (9), where 72% of the participants were concerned with the difficulty of an adequate representation to express a collective opinion. Another risk 66% of the KCE participants identified was the risk of subjectivity, which was another barrier highlighted by interviewees in the current study, expressing the concern that patients and disease specific patient organizations during the reimbursement discussions would only speak from their own perspective and their disease, whereas reimbursement decisions also impact healthy citizens and patients in other disease areas. In the UK, an interview-based qualitative study by Staley et al. (25) with members of the *National Institute for Health and Care Excellence* (NICE)^15^ showed that patient involvement through the provision of patient statements complementary to the clinical and economic evidence enables Health Technology Assessment (HTA) representatives to consider the evidence in a different light. However, they describe as a main barrier to using written statements the misperception that these statements are a form of evidence, when in fact they are experiential knowledge.

Taking interview and survey results together regarding the value of patient involvement in the lifecycle of medicines, an alignment can be perceived; survey participants acknowledged the importance of including patient preferences throughout in the lifecycle of medicines, which is in line with the various opportunities and benefits interviewees described related to patient involvement. The importance survey participants attributed to including patient preferences is also reflected in their willingness to participate in preference studies, as the majority of survey participants indicated to be willing to participate in preference studies to “improve development of treatments,” because “it is important to explore and listen to patient preferences” and “to have a voice as patients”. Comparing the responses from hospital visitors to those of patient representatives, it seems that both groups found it important to involve patients in the early and clinical stages of development. This is reflected by the importance given to including patient preferences in each phase of the life cycle by hospital visitors and the opportunities that patient representatives saw for patient involvement in the early and clinical stages of development. Regarding reimbursement specifically, it is striking that although hospital visitors, including patients, found it important to include patient preferences, interviewees, including a patient representative, were unsure about direct patient involvement during this phase and referred to barriers, such as budgetary constraints and the fact that patients and disease specific patient organizations might be biased toward wanting the lowest price. A potential explanation for this might be a differing level of familiarity with the Belgian reimbursement procedure between hospital visitors and the patient representative. Potential solutions might be, as suggested by patient representatives: (i) to involve umbrella patient organizations during the reimbursement discussion or (ii) to only involve patients for determining medical needs during the first phases of development, which could inform the “medical need” criterion for reimbursement.

### Current landscape of patient involvement in the lifecycle of medicines

Although referring to an increasing number of efforts coming from industry, regulatory and reimbursement stakeholders, interviewees labeled the level and approaches of actively involving patients as rather low and scattered across different phases of the lifecycle of medicines. Zooming in on the discovery and clinical development phase, the same held true: interviewees mentioned an increasing number of efforts of pharmaceutical industry for requesting input from patients and patient organizations on aspects, such as the administration form and relevance of the endpoints but described the level of *actively* involving patients as rather limited. Outside the Belgian context, a limited patient involvement in medicines development has been described by others (2, 5, 26) and seems to be in accordance with the findings of the study by Lowe et al. (1), where interviewees could only share few instances where medicines development was aligned according to the needs of patients. Recent examples however demonstrate that active patient involvement in early development increases; some patients' parents start their own pharmaceutical company to speed up R&D of medicines for the disease affecting their child^16^.

Interviewees in this study indicated that the involvement of patients during the Belgian marketing authorization procedure in practice consists out of the presence of a patient representative in the commission tasked with evaluating clinical trials and advising the minister on the national marketing authorization. However, their role in this commission is advisory^17^. According to interviewees, patient involvement in pricing is currently sometimes operationalized through lobbying by patient organization and indirect studies preceding the pricing decision. As to Belgian reimbursement, there was the general view among interviewees in this study that patients are currently not requested to provide direct input during the reimbursement discussion. The latter finding aligns with the composition of the Belgian Medicines Reimbursement Committee^18^. Moreover, in the present study, some interviewees mentioned that in the Belgian reimbursement procedure, the health insurance funds *try* to represent the public, including patients. This finding was also found in the Delphi panel, conducted by the KCE (9), concluding that health insurance funds “*were considered to be the stakeholder de facto representing the citizens and patients in the decisional process today*.” Taking interview and survey results together, an alignment can be perceived regarding the current level of patient involvement in the lifecycle of medicines, as most survey participants thought that patient preferences were used little or moderately across the different phases of the lifecycle of medicines.

### Bridging the gap between theory and practice: a framework for action

Despite present barriers, as indicated by interviewees in this study, the authors support investigating how patients' perspectives could improve medicines development and evaluation. The authors therefore propose a framework for action to address the following barriers (Table 4): (i) how to operationalize patient involvement, (ii) who to involve, (iii) a perceived lack of patient knowledge and (iv) regulatory compliance aspects.

**Table 4:** Barriers related to patient involvement in the lifecycle of medicines (red), suggested actions by the authors to overcome them and examples of efforts related to these actions (green).

**Barrier and explanation**	**Suggested actions**	**Examples of efforts related to suggested actions**
**How to operationalize patient involvement:** unclear how patient involvement could be practically implemented in discovery, clinical development, reimbursement and post-marketing	**Direct patient involvement** • Developing processes and tools that allow to actively involve patients e.g., regulatory guidelines stipulating: (1) for which types of development decisions industry should approach patients, (2) when and how industry should approach patients and (3) what benefit this would give for industry on the level of marketing authorization or reimbursement • Providing clear (regulatory) incentives toward industry of involving patients, such as easier market access • Research into practices of other countries on how they involve patients in their national decision-making procedures **Indirect patient involvement** • Assessing the value and limitations of preference methods e.g., by validating the results generated by different preference methods by replicating preference studies using different methods • Research aiming to develop a practical framework on how patient preferences can be implemented in decision-making throughout the lifecycle of medicines in a structured way	**Direct patient involvement** • PARADIGM project^a^ aiming to develop a framework for patient engagement in the lifecycle of medicines • The *Institute for Quality Efficiency in Health Care* (IQWiG)^b^ allows patients to take part in discussions on patient relevant outcomes^c^ • The SCOPE project^d^ aimed to strengthen the European and global pharmacovigilance network by focusing on side effect reporting by patients • The WEB-RADR project^e^ aims to develop a mobile application enabling patients to report side effects and investigates the potential of using social media in identifying safety issues • In the Netherlands, patients can report side effects directly to *The Netherlands Pharmacovigilance Centre Lareb* since 2003 (21). Patients can report on the Lareb website and recently also via a mobile application^f^ **Indirect patient involvement** • PREFER project^g^ aiming to establish recommendations about measuring and using patient preferences for informing decisions • EMA (27) and IQWiG (8, 28) pilot studies assessing the value of patient preference methods for marketing authorization and reimbursement decisions
**Who to involve:** unclear which patients or patient organizations (disease specific vs. umbrella patient organizations) should become involved in discovery, clinical development, reimbursement, post-marketing	**Direct patient involvement** • Research into practices from other countries well-advanced in the area of patient involvement • Research into what type of patient representation is needed from a decision-makers' point of view **Indirect patient involvement** Research into sample adequacy for patient preference studies finally aimed at formulating specific guidance including sample requirements for patient preference studies aiming to inform decisions e.g., conducting a patient preference study in a large and heterogeneous patient sample to determine the impact of sample characteristics on measured patient preferences or replicating preference studies within a different disease population	**Direct patient involvement** IQWiG allows patients to participate in discussions on patient relevant outcomes. Appointed patient organization representatives may submit comments on IQWiG's scientific recommendations^h^ **Indirect patient involvement** In the context of marketing authorization of medical devices, FDA describes in its guidance factors to consider regarding sample representativeness for patient preference studies (13)
**Patient knowledge and education:** unclear whether patients have sufficient knowledge and competences to contribute	**Direct patient involvement** Organizing training and education opportunities for patients e.g., organizing courses about drug development and evaluation tailored toward patients and patient organizations for preparing them to give input to industry and decision-makers **Indirect patient involvement** Testing and ensuring patient comprehension of the study goals and questions in patient preference studies e.g., (1) testing, via replicating questions in the questionnaire or inserting open questions allowing patients to give feedback about the understandability of the questions or via piloting the questionnaire (2) ensuring, via inserting interactive tools or clear and concise explanations about the terminology used in the questionnaire	**Direct patient involvement** • EURORDIS learning initiatives^i^ for patient representatives • EUPATI's video library^j^ and guidance aimed at a lay public and facilitates patient education **Indirect patient involvement** FDA guidance on how to ensure participant comprehension in patient preference studies (13)
**Regulatory compliance aspects:** direct contact between pharmaceutical companies and patients is perceived difficult due to regulatory and ethical constraints	**Direct patient involvement** • Review of regulatory frameworks to understand what exact type of interaction is prohibited • Clear (regulatory) guidance stipulating the conditions and requirements for interaction between pharmaceutical companies and patients	**Direct patient involvement** • EFPIA's code of practice to ensure relationships between pharmaceutical industry and patient organizations are ethical and transparent^k^ • UCB's framework for compliant patient engagement (29) • Roche's good practice guidelines for interacting with patient organizations^l^

a *https://imi-paradigm.eu/*.

b *IQWiG is the independent scientific institute that examines the benefits and harms of medical interventions for patients and provides recommendations for reimbursement decisions in Germany: https://www.iqwig.de/en/participation/contributing-the-patients-perspective.3070.html*.

c *Patient relevant outcomes can be defined as any outcome that it is relevant to or valued by a patient (http://www.pcori.org/establishing-definition-patient-centered-outcomes-research)*.

d *http://www.scopejointaction.eu/aims/*.

e *https://web-radr.eu/about-us/*.

f *https://www.lareb.nl/en/*.

g *http://www.imi-prefer.eu/about/*.

h *https://www.iqwig.de/en/participation/contributing-the-patients-perspective.3070.html and https://www.iqwig.de/en/about-us/institute-structure/bodies-and-committees.2957.html*.

i *https://www.eurordis.org/patient-empowerment-and-training*.

j *https://www.eupati.eu/*.

k *https://www.efpia.eu/media/24310/3c_efpia-code-of-practice-on-relationships-pharmapluspt-orgs.pdf*.

l *https://www.roche.com/dam/jcr:43ceebf6-1d7d-4305-a24c-16331fdbe4ba/en/guidelines_on_working_with_patient_groups.pdf*.

*Suggested actions and related examples are categorized depending on whether they relate to: i) direct patient involvement, via the participation of patients (or patient organizations) in discussions, or ii) indirect patient involvement, via the measurement and inclusion of patient perspectives on the value of health innovations via patient preference studies*.

The future seems bright; several projects have been initiated to address these barriers. Examples of ongoing European efforts include PREFER^19^, PARADIGM^20^, EUPATI^21^ and WEB-RADR^22^ (Table 4). It is striking that despite all of these ongoing and past efforts, interviewees and survey participants still labeled the level of current patient involvement as rather low and none of these efforts nor projects were mentioned by interviewees, which could imply that: (i) interviewees and survey participants were not familiar with these efforts and/or (ii) the impact of these efforts has yet to be reflected in actual and visual changes in current decision-making and development procedures. This study therefore not only underscores the importance of the successful continuation of these efforts, it also points toward the necessity of familiarizing and educating stakeholders (including patients, patient organizations, academics, healthcare practitioners, payers and industry) with the concept and potential of patient involvement and patient preferences in the lifecycle of medicines, e.g., through the organization of webinars on these topics^23^.

The present study also reveals tension around the question of whether to involve individual patients, disease-specific patient organizations or umbrella patient organizations in decision-making. Whereas, direct involvement would give decision-makers a quick and in-depth idea of individual patient experiences, its ability of representing the entire patient population was criticized by interviewees in the current study and has also been criticized by other authors (12, 17, 30–37). Patient organization representatives could be useful in this regard since they are representing a group of patients rather than one patient, but as mentioned by interviewees in the current study, they could be criticized for being dependent of pharmaceutical companies. Moreover, then the question arises of which patient organization to involve. A potential solution to address these issues might be, as suggested by some interviewees and several authors (10, 17, 27, 31, 33, 34, 36, 38–42), the use of evidence from (quantitative) patient preference studies, by which patient preferences are being measured from a group of patients. To decide whether the input from disease-specific or umbrella patient organizations is needed, the authors propose to address this problem bottom-up, via looking at what type of patient information is needed per decision. When a decision is taken about a disease-specific issue, e.g., decisions concerning the feasibility of a clinical trial protocol and for determining the relevance of endpoints, it could be more appropriate to use input from disease-specific patient organizations. Conversely, when decisions need to be taken from a broader perspective and requires input from neutral parties, e.g., for pricing and reimbursement discussions, then it could be useful to involve umbrella patient organizations. This choice seems to also depend on the stage of the drug life cycle, i.e., the decision-making context, and its affected stakeholder population. For example, if the decision affects a specific patient population, e.g., for endpoint determinations or marketing authorization, it might be more appropriate to ask individual patients or disease specific patient organizations. In contrast, when the decision affects a broader population, e.g., for reimbursement decisions taken in a country with a publicly funded healthcare system, then it might be more appropriate ask input from umbrella patient organizations.

Interviewees in the present study suggested the use of methods that indirectly capture patients' perspectives to overcome the barrier of difficult direct contact between industry and patients and to help answering the questions on how to operationalize patient involvement and which patients to involve. The authors therefore recommend further exploration and validation of qualitative and quantitative methods that enable the measurement of patient preferences, which is one of the aims of PREFER^24^.

### Limitations

Regarding the interviews, interviewees were selected based upon purposive sampling and an initial sample of potential interviewees consisted out of persons suggested by the co-authors. The possibility that purposively sampling interviewees may have biased our results cannot be excluded. Further, interviewees' willingness to participate might be inherently connected to their perspectives on the involvement of patients in in the lifecycle of medicines. It was assumed, however, that using a purposively drawn sample would lead to recruiting interviewees familiar with the topic and that knowledge on the topic would help providing the research with more depth.

It was sought to include interviewees with different backgrounds and affiliations to increase the heterogeneity of the sample. However, time restrictions precluded the possibility of setting quota per stakeholder group, resulting in different amounts of interviewees per stakeholder group and a small number of interviewees per stakeholder group, both of which may have posed bias in the results. The interviewed sample consisted of 35% industry interviewees, as opposed to the other stakeholder groups that were represented in ranges between 4 and 22%. Sixty percent of patient organization representatives was affiliated with rare disease patient organizations, which may have placed a greater focus on the importance of patient involvement in these disease areas in the results. Further, although persons with positions within the Belgian (FAMHP) or European regulatory agency (EMA) were invited to participate in an interview, we did not succeed in recruiting interviewees with positions within these institutions. Therefore, the interview results concerning the current level, opportunities and barriers related to patient involvement in marketing authorization only describe the perspectives of interviewees outside the agencies responsible for marketing authorization. Similarly, persons from the Belgian “*Federal Agency for Food Chain Security*^25^” and the “*Scientific Institute of Public Health*^26^” were invited but did not respond or declined participation.

At the beginning of the interview itself, no definition of patient involvement nor patient preferences was given, which probably yielded differences in interviewees' conceptualizations of these terms. This limitation was also described in a qualitative study conducted by Utens et al. (43) focusing on Dutch stakeholders' perspectives toward incorporating patient preferences in reimbursement, stating that in their study “*respondents' conceptualizations of preferences colored their responses to the other questions. Therefore, different respondents may not have been referring to the same issues in their responses and what they meant was sometimes difficult to interpret*”. The confusion in terminology related to patient involvement has also been raised by Hoos et al. (5), stressing the need for a clear definition of both the terms “patient” and “involvement”. Other terms used in the interview guide of the current study that may have been better explained are the terms “market authorization” and “pharmacovigilance”. “Market authorization” could have been explained by providing a definition or by referring to “benefit-risk assessment”. “Pharmacovigilance” could have been explained by providing a definition e.g., “the detection of side effects”. However, the pilot interviews did not reveal that interviewees faced difficulties with understanding the questions. Furthermore, interviewees in the current study always provided enough context when making statements surrounding patient involvement, patient preferences, marketing authorization and pharmacovigilance, which is why we did not encounter difficulties in the interpretation of interviewees' responses. We agree on these points with Hoos et al. (5) and Utens et al. (43) that the differences of conceptualizations used for patient preference and involvement related terminology in practice forms an important issue in itself and we therefore also support any effort to streamline terminology surrounding patient involvement, empowerment, participation, preferences and other inter-related terms.

The interviews were not conducted one-on-one, but instead by three researchers, which might have caused that interviewees felt somewhat intimidated during the interview. Yet, this approach was considered beneficial for the analysis of the interviews, as being present during the interviews facilitated the familiarization stage of the analysis for the interviewers. However, the last two stages of the framework analysis were carried out by a researcher that was not present during the interviews. Therefore, this researcher needed to compensate by familiarizing with the interviews by thoroughly re-reading the interviews and re-listening the audio files whenever the transcript was unclear.

Concerning the surveys, although initially aimed at learning about *patients'* perspectives toward patient preferences in the lifecycle of medicines, only 35% of the surveyed sample stated having a disease. Potential reasons why the remainder of the surveyed sample did not mention a disease might be because these participants: (i) were healthy, (ii) preferred to not share this information or (iii) were not visiting the hospital for an issue concerning their own disease. In retrospect however, we feel that this sample composition might not have been a limitation *per se*, since also healthy people can become patients, since informal caregivers' perspectives are often linked to patients' perspectives and finally because informal caregivers' perspectives are valuable sources of information on itself. We therefore consider this survey as a way to learn about the public perspective toward involving patients in the lifecycle of medicines.

Other limitations of the survey relate to terminology. First, although the term “patient preferences” was explained^27^ at the beginning of the survey, the answers to the question on whether survey participants had already participated in a preference study demonstrated that some survey participants confused the term “patient preference study” with the term “clinical trial”. This confusion might also suggest that these survey participants did not completely understand the concept of patient preferences when filling out the entire survey. A second limitation concerning terminology relates to the various terms used to denote the different phases of the lifecycle of medicines^28^ in the survey question on the current level of use of patient preferences during the different phases of the lifecycle of medicines. Although these terms were phrased as easy as possible to understand for a non-expert audience and survey participants had the possibility to ask questions when facing difficulties during the completion of the survey, it remains unknown whether survey participants actually understood these terms when answering this question.

Concerning both the interviews and surveys, it is important to note that since no statistical testing was performed, both the interview and survey results are to be interpreted mainly in a qualitative manner. Further, both the interview and survey results are not to be interpreted as being generalizable to a larger population than the interviewed or surveyed sample, respectively. For example, the survey results only reflect the views of the surveyed hospital visitors in the UZ Leuven. Second, only Dutch speaking persons were included in this study, which may have posed a selection bias. A third limitation concerning both the interviews and survey relates to the terminology used in the survey questionnaire vs. the interview guide. Throughout the questions of the interview guide, the terms “patient involvement” and “patient preferences” were used interchangeably whereas in the survey, the term “patient preferences” was explained^29^ at the beginning and used throughout the survey. This might explain why interview results mainly concern the opinions of Belgian stakeholders toward aspects of patient involvement whereas survey participants thought about the importance and current level of incorporating *patients' decisions and choices* about their own healthcare and treatment when filling in the survey.

### Conclusions

This study provides an in-depth understanding of the current landscape, opportunities and barriers related to patient involvement in the lifecycle of medicines according to Belgian stakeholders. Both survey participants and interviewees indicated that the current level of patient involvement in the lifecycle of medicines is rather limited. The main opportunities to involve patients in development highlighted by interviewees were during early development, when decisions are made on which treatments to develop and during clinical development, when decisions are made on selection of endpoints and clinical trial protocols. In addition, the majority of survey participants indicated to be willing to contribute to these activities through participation in patient preference studies. Despite these opportunities, questions surrounding patient knowledge, how and which patients to involve represent important barriers toward practical implementation of patient involvement in the lifecycle of medicines. The barriers identified in this study hamper transitioning patient involvement from theory to practice. Bridging this gap will require addressing the identified barriers and unresolved questions surrounding the right methodology to involve patients, identification of the “right patients” to involve and means to increase patient knowledge. In order to do so, further research should focus on assessing the value of methods that allow to indirectly capture patients' perspectives both in the context of the development as in the context of evaluation of medicines.

## Ethics statement

Interviews were carried out in accordance with the recommendations of the Ethics Committee of the Faculty Pharmaceutical Sciences at the University of Leuven, Belgium (reference ethical dossier: mp12721), with written informed consent from all interviewees. All interviewees gave written informed consent in accordance with the Declaration of Helsinki. After consulting the Medical Ethics Committee UZ KU Leuven/Research and in view of the fact that data from the surveys was collected and analyzed anonymously, written informed consent for the surveys was deemed unnecessary.

## Author contributions

All authors provided substantial contributions to the conception and design of the work. LV, LM, and CC collected the data. RJ, EvO, LV, LM, and CC were involved in the interpretation of data for the work. RJ and EvO drafted the work and the remainder of the authors (LV, LM, CC, KP, MD, HS, SS, and IH) revised it critically for important intellectual content. All authors approved the final version to be published and are all accountable for all aspects of the work in ensuring that questions related to the accuracy or integrity of any part of the work are appropriately investigated and resolved.

### Conflict of interest statement

The authors declare that the research was conducted in the absence of any commercial or financial relationships that could be construed as a potential conflict of interest.

## Footnotes

^1^http://www.imi-prefer.eu/about/.

^2^https://imi-paradigm.eu/.

^3^https://www.fda.gov/Drugs/NewsEvents/ucm582081.htm.

^4^http://www.ema.europa.eu/docs/en_GB/document_library/Other/2009/12/WC500018013.pdf.

^5^http://www.ema.europa.eu/ema/index.jsp?curl=pages/audience/alp_audiencetype_000001.jsp&mid= and http://www.ema.europa.eu/docs/en_GB/document_library/Report/2018/06/WC500251085.pdf.

^6^http://www.adrreports.eu/.

^7^The term “patient involvement” was not defined at the beginning of the interview but referenced to in the interview questions as “patients being involved” or “having a say” in the lifecycle of medicines (Supplemental Material 1).

^8^The term “patient preferences” was not defined at the beginning of the interview.

^9^A lay definition for “patient preferences” was given at the beginning of the survey: “patients' decisions and choices about their own healthcare and treatment based on what they consider important in terms of comfort, well-being, lifestyle or financial preferences” (Supplemental Material 3).

^10^One interviewee preferred to take part together with one extra interviewee, resulting in a total amount of 23 interviewees and 22 interviews.

^11^In the survey, “a study that measures patient preferences” was used to refer to a patient preference study (Supplemental Material 3).

^12^A lay version of the definition for “patient preference information” by the FDA (“qualitative or quantitative assessments of the relative desirability or acceptability to patients of specified alternatives or choices among outcomes or other attributes that differ among alternative health interventions”) was given at the beginning of the survey: “patients' decisions and choices about their own healthcare and treatment based on what they consider important in terms of comfort, well-being, lifestyle or financial preferences” (Supplemental Material 3).

^13^*Amyotrophic Lateral Sclerosis, a rare neurological disorder*.

^14^Background on the Soliris® case, adapted from Picavet et al. (16): In 2013, the reimbursement of Soliris® was refused. Controversy arose when the case of a patient was shown in the media. Afterwards, it became clear that the pharmaceutical company deliberately brought attention to this case in the media with the purpose of changing the reimbursement decision.

^15^HTA body supporting payer decisions in the UK.

^16^https://globalgenes.org/raredaily/183432-2/.

^17^https://www.fagg-afmps.be/nl/Commissies/commission_pour_les_medicaments_a_usage_humain_cmh_.

^18^The Belgian Medicines Reimbursement Committee is the committee that acts as an advisory body for the Minister for Social Affairs with regard to reimbursement decision of medicines. See following link for composition of committee: https://www.inami.fgov.be/nl/riziv/organen/Paginas/commissie-tegemoetkoming-geneesmiddelen.aspx#Wat_is_de_samenstelling_van_de_CTG?.

^19^http://www.imi-prefer.eu/about/.

^20^https://imi-paradigm.eu/.

^21^https://www.eupati.eu/

^22^https://web-radr.eu/about-us/.

^23^Examples of webinars organized on patient involvement and patient preferences: 1) https://www.fda.gov/MedicalDevices/NewsEvents/WorkshopsConferences/ucm518783.htm, 2) https://www.pcori.org/sites/default/files/PCORI-MDIC-Ambassadors-Webinar-Presentation-Slides-072016.pdf 3) http://iahpr.org/videos-on-health-preference-research/ https://www.youtube.com/watch?v=yi22pUa1aBU, 4) https://www.imi-prefer.eu/events/event/?eventId=33454.

^24^http://www.imi-prefer.eu/about/.

^25^The Federal Agency for Food Chain Security is the agency in Belgium responsible for all verifications with regard to food safety.

^26^The Scientific Institute of Public Health performs scientific research in the fields of public health, animal health and food safety.

^27^The term patient preferences was defined at the beginning of the survey as “patients' decisions and choices about their own healthcare and treatment based on what they consider important in terms of comfort, well-being, lifestyle or financial preferences”.

^28^The survey question was as follows (originally in Dutch): “To what extent do you think that patient preferences are currently being measured and taken into account at the different stages of the life cycle of a medicine or medical device? The phases of the lifecycle of medicines: research and development, testing on animals, testing on people, approval for coming onto the market, price determination, reimbursement, pharmacovigilance”.

^29^The term patient preferences was defined at the beginning of the survey as “patients' decisions and choices about their own healthcare and treatment based on what they consider important in terms of comfort, well-being, lifestyle or financial preferences”.
