# Supplementary material for: Patient Involvement in the Lifecycle of Medicines According to Belgian Stakeholders: The Gap Between Theory and Practice
Source: Front Med (Lausanne). 2018 Oct 11;5:285. doi: 10.3389/fmed.2018.00285 (PMC6193089; doi:10.3389/fmed.2018.00285)
Supplement: Supplementary file 1 [file Data_Sheet_1.docx]

Supplementary Material

Patient Involvement in the Lifecycle of Medicines according to Belgian Stakeholders: The Gap between Theory and Practice

**Rosanne Janssens*, Eline van Overbeeke, Lotte Verswijvel, Lissa Meeusen, Carolien Coenegrachts, Kim Pauwels, Marc Dooms, Hilde Stevens, Steven Simoens, Isabelle Huys**

*** Correspondence:** rosanne.janssens@kuleuven.be

# Interview guide^[[1]](#footnote-1)^

1. How important do you think it is that patients are involved in the drug lifecycle?
   1. Examples: in the development of a new drug, clinical studies, market authorization, pricing, reimbursement and pharmacovigilance
   2. Why?
2. In your opinion, to what extent do patients already have a say in the different phases within the drug lifecycle?
3. How is the implementation of patient preferences currently according to you?
   1. How are these preferences currently measured?
4. At which stages of the drug lifecycle can patient preferences offer added value?
   1. In what way?
5. Why could patient preferences be important for a pharmaceutical company?
6. Why could patient preferences be important for the NIHDI^[[2]](#footnote-2)^ or the FAMHP^[[3]](#footnote-3)^?
7. Why could patient preferences be important for healthcare professionals?
   1. Examples: doctors, pharmacists, nurses, specialists and physiotherapists
8. Can the current approach of pharmacovigilance be further improved?
   1. In what way would this be best?
   2. What is the role of the patient in this?

# Framework matrix^[[4]](#footnote-4)^


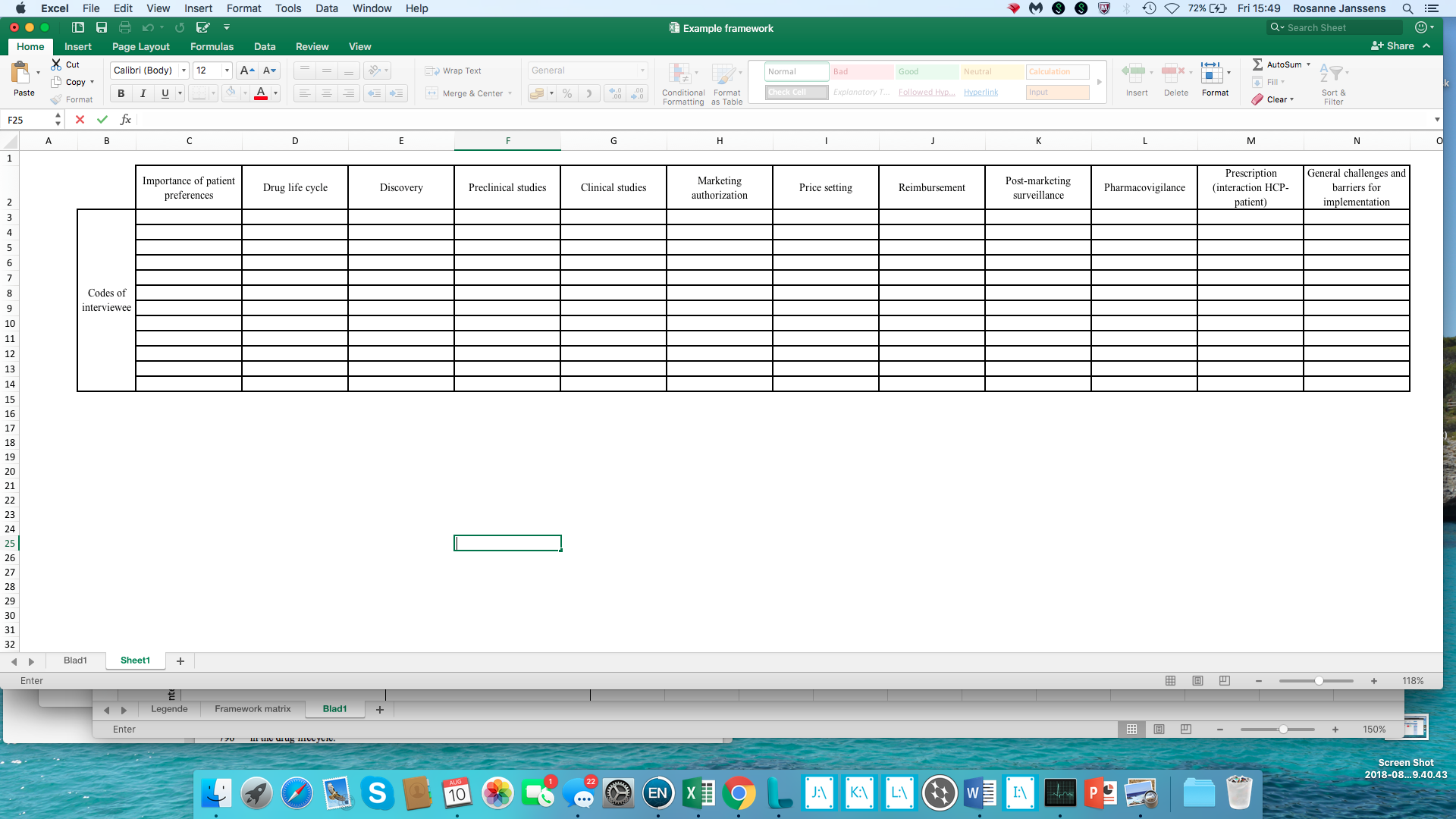


# Survey^[[5]](#footnote-5)^

|  | Sex:   - Male - Female   Age:  Nationality:  Country of origin:  Disease:  Treatment (medication):  **Explanation preferences of patients:**  Patients' decisions and choices about their own healthcare and treatment based on what they consider important in terms of comfort, well-being, lifestyle or financial preferences. | | | | | | | | |
| --- | --- | --- | --- | --- | --- | --- | --- | --- | --- |
| 1. | How important do you think that patients' preferences are measured and taken into account when researching and developing new medicines or medical devices (e.g pacemaker, blood pressure monitor, thermometer, ...)? | | | | | | | | |
| o | Very important | | o Important | | o Not important | | | o Not important at all | |
| 2. | How important do you think that patients' preferences are measured and taken into account when testing and improving medicines in clinical trials? (clinical study = studies of efficacy, side effects, ... of drugs on volunteers and patients) | | | | | | | | |
| o | Very important | | o Important | | o Not important | | | o Not important at all | |
| 3. | How important do you think that patient preferences are measured and taken into account when approving new medicines or medical devices to bring them to the market? | | | | | | | | |
| o | Very important | | o Important | | o Not important | | | o Not important at all | |
| 4. | How important do you think that patient preferences are measured and taken into account when setting the price of new medicines? | | | | | | | | |
| o | Very important | | o Important | | o Not important | | | o Not important at all | |
| 5. | How important do you think that patients' preferences are measured and taken into account in decisions taken by the government regarding reimbursement of medicines and medical devices? | | | | | | | | |
| o | Very important | | o Important | | o Not important | | | o Not important at all | |
| 6. | How important do you think that patient preferences are measured and taken into account in pharmacovigilance of medicines? (Pharmacovigilance or pharmacovigilance: the task of monitoring the safety of medicines and ensuring that the risks of a medicine do not exceed its benefits) | | | | | | | | |
| o | Very important | | o Important | | o Not important | | | o Not important at all | |
| 7. | To what extent do you think that patient preferences are currently being measured and taken into account at the different stages of the life cycle of a medicine or medical device?  The phases of the drug lifecycle:   - Research and development - Testing on animals - Testing on people - Approval for coming onto the market - Price determination - Reimbursement - Pharmacovigilance | | | | | | | | |
| o | Too much | o A lot | | o Sufficient | | | o Little | | o Too little |
| 8. | Have you already participated in a study that measures patient preferences? | | | | | | | | |
| o | Yes | | | | | o No | | | |
|  | If yes: In what way were your preferences measured:   - Multiple choice questionnaire - Interview - Other:   _______________________________________________________________________  Who organized this study?  _______________________________________________________________________ | | | | | | | | |
| 9. Would you as a patient be prepared to actively participate in a study that measures patient preferences? | | | | | | | | | |
| o Yes | | | | | | o No | | | |
| Why (not)?  _______________________________________________________________________  If yes: In what way do you see this happening?  _______________________________________________________________________ | | | | | | | | | |
| 10. Do you have anything to add?  _______________________________________________________________________ | | | | | | | | | |

# Overview of interviewees’ functions

| Stakeholder group | Function(s) |
| --- | --- |
| Academic | Professor (rheumatology) |
| Academic | Professor (pharmacology) |
| Academic | Professor (nephrology) |
| Academic | Professor (rheumatology) |
| Health insurance fund | Advisory function |
| Health insurance fund | Advisory function |
| Health insurance fund | Coordination |
| Belgian reimbursement agency | Senior position |
| Pharmaceutical industry | Medical, senior position |
| Pharmaceutical industry | Medical |
| Pharmaceutical industry | Scientific officer, senior position |
| Pharmaceutical industry | Medical, senior position |
| Pharmaceutical industry | Global pricing, senior position |
| Pharmaceutical industry | Medical, senior position |
| Pharmaceutical industry | Patient engagement, senior position |
| Pharmaceutical industry | Patient care, senior position |
| Patient organization | Executive board (rare diseases) |
| Patient organization | Executive board (rare disease) |
| Patient organization | Executive board (rare disease) |
| Patient organization | Management |
| Patient organization | Management |
| Hospital pharmacist | Hospital pharmacist (orphan diseases) |
| Hospital pharmacist | Hospital pharmacist (pediatric oncology) |

1. *This is a translated version of the interview guide; the original interview guide was in Dutch.* [↑](#footnote-ref-1)
2. *The National Institute for Health and Disability Insurance National Institute for Health and Disability Insurance (NIHDI) is the drug reimbursement agency in Belgium.* [↑](#footnote-ref-2)
3. *The Federal Agency for Medicines and Health Products (FAMHP) is the Belgian competent authority responsible for the quality, safety and efficacy of medicines and health products.* [↑](#footnote-ref-3)
4. *Simplified version of the final framework matrix developed in stage 4 of the framework analysis. The interviewee codes were mentioned in the x-axis and the codes in the y-axis. The empty cells contained summaries of transcripts and literal quotations.* [↑](#footnote-ref-4)
5. *This is a translated version of the survey, the original survey was in Dutch.* [↑](#footnote-ref-5)
